# Comparison of the Partial Structure and Antioxidant Activity of Polysaccharides from Two Species of Chinese Truffles

**DOI:** 10.3390/molecules25184345

**Published:** 2020-09-22

**Authors:** Xiaolin Li, Zhongkai Zhu, Lei Ye, Zongjing Kang, Xiaoping Zhang, Yue Huang, Bo Zhang, Yuanfeng Zou

**Affiliations:** 1 Soil and Fertilizer Institute, Sichuan Academy of Agricultural Sciences, Chengdu 610066, China; kerrylee_tw@sina.com (X.L.); yeleidewangyi@163.com (L.Y.); catherinekang@163.com (Z.K.); xiaopingzhang97@163.com (X.Z.); yuehuang@stu.sicau.edu.cn (Y.H.); bozhang5658@foxmail.com (B.Z.); 2 Natural Medicine Research Center, College of Veterinary Medicine, Sichuan Agricultural University, Chengdu 611130, China; zhongkaizhu6@163.com; 3 Department of Microbiology, College of Resources, Sichuan Agricultural University, Chengdu 611130, China

**Keywords:** truffle, *Tuber panzhihuanense*, *Tuber pseudoexcavatum*, polysaccharides, structure

## Abstract

Truffles are world-renowned premium commodities. Due to their unique aroma and rarity, the price of truffles has always been very high. In this study, Diethylaminoethyl anion exchange chromatography and gel filtration were employed for polysaccharide purification from two different species of Chinese truffles. Three polysaccharide fractions were obtained from *Tuber panzhihuanense* and referred to as TPZ-NP, TPZ-I, and TPZ-II. Additionally, two polysaccharide fractions were purified from *T. pseudoexcavatum* (TPD-NP and TPD-I). The results of structural elucidation indicated that the polysaccharide from different species showed different monosaccharide composition and linkage units, as well as molecular weight. Two of the polysaccharide fractions with the highest yield, TPZ-I and TPD-I, were chosen for biological testing. The results indicated that both fractions displayed antioxidant properties through mediation of the intestinal cellular antioxidant defense system, which could protect cultured intestinal cells from oxidative stress-induced damage and cell viability suppression. The TPD-I fraction showed stronger antioxidant effects, which may be due to the difference in structure. Further study on the structure-activity relationship is needed to be done.

## 1. Introduction

The truffle, the hypogeous comycota fungus, has been found around the world. There are more than 100 species, and they grow as commensal ectomycorrhiza on plant roots [1,2]. Due to severe growing conditions and low productivity, truffles are high-price commodities. Truffles are mainly distributed in Europe; however, Chinese truffles, widely known as Asian truffles, are also consumed [3,4]. The Chinese truffle is considered an edible and medicinal fungus in China, and it is mainly found in the Sichuan and Yunnan Provinces. In China, a large number of black truffles are distributed, and more than 20 species of the *Tuber* genus have been identified in China since 1985 [5,6,7]. Black truffle is the main member of the *Tuber* genus, and *T. melanosporum* is the major species in black truffle. It is known colloquially as “underground gold” due to its peculiar aroma and taste, which give it higher economic value than other black truffle species [8,9]. In 1998, a new black truffle species was discovered in Sichuan, China and named *T. pseudoexcavatum* [10]. Its ascocarps are subglobose with spinose-reticulate ornamentation, and it is buried deep in the earth [11]. In recent years, due to the good quality and aroma of Chinese truffles, the market price has gradually increased, and their position in the truffle market has become increasingly more prominent.

Truffles are regarded as “edible diamonds” because of their high nutritional value. They contain amino acids, minerals, proteins, carbohydrates, and fats [12]. It has been reported that there are high amounts of polysaccharides in truffle fruit bodies, ranging from 1.52–5.23% in different truffle species [13,14,15]. Moreover, these polysaccharides exhibit many biological activities. A water-soluble heteroglycan from an edible truffle *T. rufum* exhibited antioxidant activity [16]. Chinese scholars isolated and separated many polysaccharide components from different truffles, including *T. sinense*, *T. himalayense*, and *T. indicum*, and these polysaccharides displayed excellent anti-tumor activity [17]. However, there are few reports regarding carbohydrates from *T. panzhihuanense* and *T. pseudoexcavatum.* Thus, it is interesting to inquire about the polysaccharides from these two truffle species.

Oxidation is essential for many organisms to generate energy and support the metabolism of aerobic cells. However, in some cases, uncontrolled metabolic pathways can generate free radicals. When the generation of oxygen free radicals exceeds the scavenging ability of the antioxidant system, it will cause damage to cells or tissues, known as oxidative stress [18,19]. Truffles have a high content of antioxidants and phenolic compounds, and exhibit antioxidant activities. The antioxidant-conferring agents were catechin, ferulic acid, p-coumaric acid, cinnamic acid, and polysaccharides [12,20]. In recent reports, truffle polysaccharides were identified as natural antioxidants that have essentially no side effects compared to synthetic antioxidants [6,21,22]. Given that most of the polysaccharides cannot be degraded by mammalians but by intestinal microbiota, it is reasonable for the intestine to be the major target organ of polysaccharides [19,23]. However, few studies have shown the effects of truffle polysaccharides on intestinal oxidative stress. Therefore, this study was focused on the effects of truffle polysaccharides on oxidative stress in intestinal epithelial cells.

In this paper, we isolated the polysaccharide fractions from the fruiting bodies of two Chinese truffles, *T. pseudoexcavatum* and *T. panzhihuanense.* Crude water extracts and five polysaccharide fractions were obtained. Structural analysis of the polysaccharide fractions was also performed by gas chromatography-mass spectrometry (GC-MS), and the antioxidant activities of two acidic polysaccharide fractions with the highest yields were also investigated in vitro.

## 2. Results and Discussion

### 2.1. The Carbohydrate, Phenol, and Protein Content of Tubers Extracts

The yields of crude water extracts from *T. pseudoexcavatum* (TPD) and *T. panzhihuanense* (TPZ) were 3.93% and 6.44%, respectively. As shown in Table 1, TPZ was richer in proteins, phenols, and carbohydrates than TPD. The two truffles were both rich in carbohydrates, and phenol was the second most abundant component. Through this extraction method, proteins were the least abundant in the crude fractions.

### 2.2. Fractionation of Polysaccharides from Tubers

**Figure 1:**
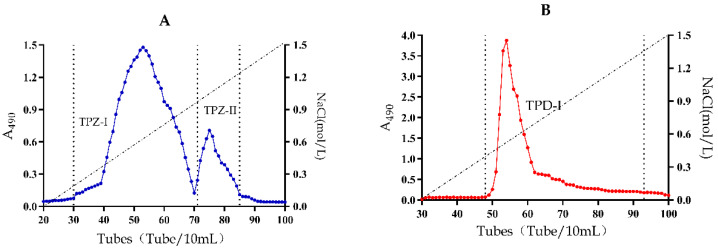
The carbohydrate elution profiles monitored by the phenol-sulfuric acid assay. (**A**) Ion-exchange chromatography elution profile of crude polysaccharide from fruit bodies of *T. panzhihuanense*. (**B**) Ion-exchange chromatography elution profile of crude polysaccharide from fruit bodies of *T. pseudoexcavatum*.

**Figure 2:**
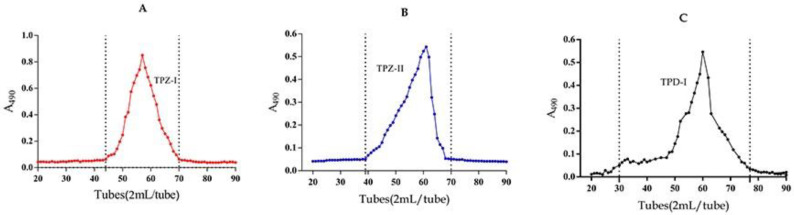
The carbohydrate elution profiles of gel filtration chromatography, monitored with the phenol-sulfuric acid assay. Elution profiles of (**A**) TPZ-I, (**B**) TPZ-II, and (**C**) TPD-I.

The crude polysaccharide (300 mg) was dissolved in 20 mL distilled water and separated by ion-exchange chromatography. One neutral fraction (TPZ-NP) and two acidic polysaccharide fractions (TPZ-I and TPZ-II) were obtained from *T. panzhihuanense*, based on the two peaks in the carbohydrate elution profile (Figure 1). Meanwhile, one neutral fraction (TPD-NP) and one acidic polysaccharide fraction (TPD-I) were purified from *T. pseudoexcavatum* (Figure 1). All of the acidic fractions (TPZ-I, TPZ-II, and TPD-I) were further purified by gel filtration (Figure 2). As shown in Figure 2, only one peak was obtained in each sample after gel filtration.

### 2.3. Molecular Weight of Polysaccharides

As shown in Table 2, the fraction TPZ-NP (5.13 kDa) had the lowest molecular weight among all the polysaccharide fractions. The molecular weight of the neutral polysaccharide was lower than the acidic polysaccharide. Moreover, the molecular weight of TPD-I was close to that of TPZ-II. The molecular weight (*Mw*) of TPZ-I (30.84 kDa) was higher than TPZ-II (17.43 kDa) and TPD-I (18.91 kDa).

### 2.4. Chemical Composition of Polysaccharide Fractions

The monosaccharide composition was determinate by methanol hydrolysis, and the results are displayed in Table 3. The neutral polysaccharide fractions from *T. panzhihuanense* and *T. pseudoexcavatum* have similar monosaccharide compositions, which were mainly mannose (Man) and rhamnose (Rha) with lower abundances of glucose (Glc) and galactose (Gal) (Table 3). TPZ-NP consisted of Rha, Gal, Man, and Glc (5:13:1:2, molar ratio), while TPD-NP contained Rha, Gal, Man, and Glc in the molar ratio of 8:26:1:4. For the acidic polysaccharide fractions, TPZ-I and TPD-I had similar compositions of monosaccharides, mainly Rha, Man, and Glc, with lower abundances of Gal and Mannuronic acid (ManA). TPD-I contained more acidic monosaccharides (1.9%) but a fewer number of total neutral monosaccharides (98.1%) than TPZ-I, in which the acidic and neutral monosaccharides accounted for 0.9% and 99.1% of the total monosaccharides, respectively (Table 3). TPZ-I contained higher amounts of Man and Rha but lower amounts of Gal and Glc than TPD-I.

These findings are similar to a previous study about the polysaccharide composition of Chinese *T. indicum*, which was also composed of mannose, glucose, galactose, and rhamnose [6]. However, the monosaccharide composition of *T. aestivum* differed from the truffle species in the present study, as it is mainly composed of mannose, glucose, and galactose. Meanwhile, it has also been reported that *T. melanosporum* polysaccharide contained the same composition as *T. aestivum* (approximate molar ratio of mannose:glucose:gal = 4:3:1) [13,17]. Six polysaccharide fractions were isolated from submerged culture of *T. melanosporum* [24]. These fractions mainly contained glucose, mannose, and galactose, and a small amount of glucuronic acid and galacturonic acid, which is quite different from our study.

### 2.5. Glycosidic Linkages

The glycosidic linkage types of these two polysaccharide fractions were quite similar to the results of GC-MS, but with different ratios (Table 4). The acidic polysaccharides from the fruit bodies of *T. panzhihuanense* and *T. pseudoexcavatum* had similar monosaccharide glycosidic linkages but were different between TPZ-I and TPD-I (Table 4). Those two types of acidic polysaccharides mainly contain Rha, Glc, and Man and a trace of Gal and ManA. Man was the most complicated monosaccharide present in the acidic polysaccharide, as it has several linkage units, such as 1→2-linked, 1→3-linked, and 1→2,6-linked. However, the glycosidic linkages were found in different abundances, especially the 1→4 linkages in glucose. The abundance of these linkages in TPD-I was nearly four times that of TPZ-I. Overall, the linkage types obtained in fractions *T. panzhihuanense* and *T. pseudoexcavatum* were similar to those of another study of a rhamno-mannan polysaccharide from *Burkholderiamultivorans* [25]. In another study, the polysaccharide fraction from *T. sinoaestivum* had similar glycosidic linkages with that in the present study [26].

### 2.6. Antioxidant Activity

#### 2.6.1. Determination of Cell Viability

**Figure 3:**
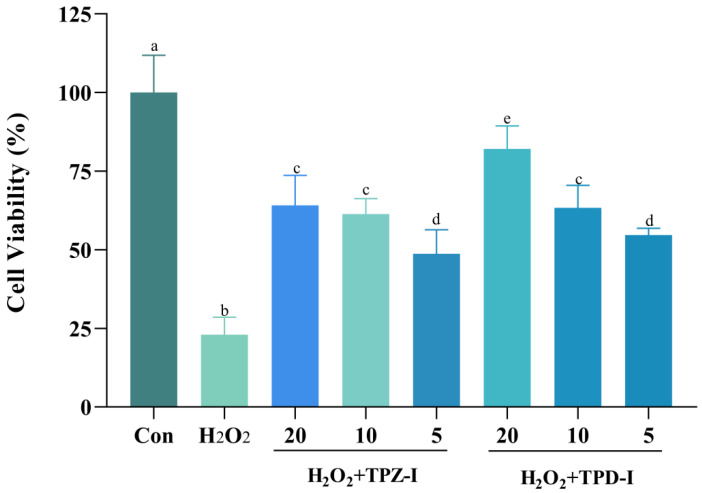
Protective effects of TPZ-I and TPD-I on viability loss in porcine jejunum epithelial cells (IPEC-J2) induced by H_2_O_2_ (0.2 mM). The concentrations of polysaccharide fractions were 20, 10, and 5 μg/mL, respectively. Values are presented as means ± Standard error. Bars with different letters are statistically significant (*p* < 0.05).

The protective effects of TPZ-I and TPD-I, regarding cell viability, are shown in Figure 3. There was a significant difference between the control and model group (200 mM H_2_O_2_). TPZ-I and TPD-I both effectively reversed the damage of H_2_O_2_ to cells. The polysaccharide of TPD-I showed higher cell viability, compared with TPZ-I. In particular, 20 μg/mL TPD-I exhibited the highest antioxidant activity and ability to improve cell viability. The fractions TPZ-NP, TPZ-II, and TPD-NP showed no obvious activity.

#### 2.6.2. Antioxidant Enzyme Activity

**Figure 4:**
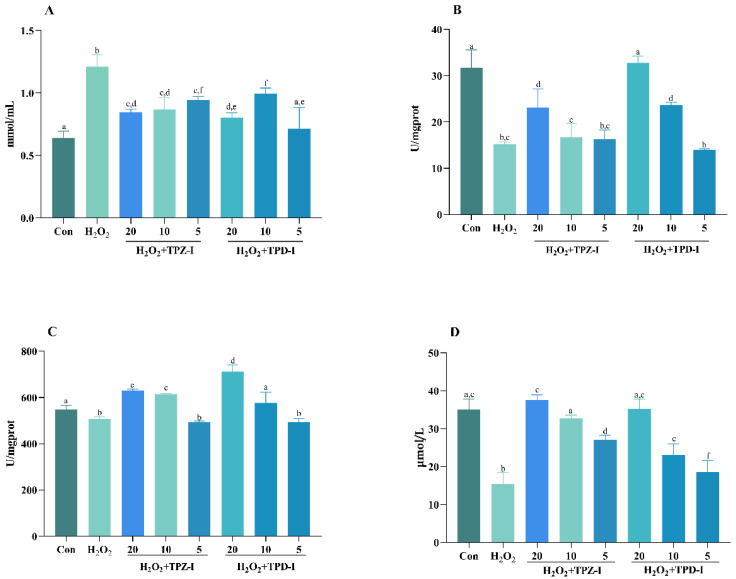
Effects of TPZ-I and TPD-I on the activities of malondialdehyde-MDA (**A**), superoxide dismutase-SOD (**B**), catalase-CAT (**C**), and glutathione peroxidase-GSH-Px (**D**) in IPEC-J2 cells. The concentrations of the polysaccharide fractions were 20, 10, and 5 μg/mL, respectively. Values are presented as means ± SD (*n* = 6). Bars with different letters are statistically significant (*p* < 0.05).

The effects of TPZ-I and TPD-I on the activities of malondialdehyde (MDA), superoxide dismutase (SOD), catalase (CAT), and glutathione peroxidase (GSH-Px) in porcine jejunum epithelial cells (IPEC-J2) cells are shown in Figure 4. From Figure 4A,C, the MDA level significantly increased after 20 μmol/mL H_2_O_2_ treatment. However, two acidic polysaccharides, TPZ-I and TPD-I, significantly reduced the MDA level. In particular, 20 μg/mL TPD-I reduced the production of MDA (*p* < 0.05). In addition, SOD activity significantly decreased, and the two acidic polysaccharides reversed the effects of H_2_O_2_. The TPD-I improved SOD activity the most (*p* < 0.05). Moreover, 20 μg/mL TPD-I significantly increased the secretion levels of CAT and GSH-Px in cells (*p* < 0.05). More specifically, the effect of TPD-I on CAT activity was greater than TPZ-I (20 μg/mL and 10 μg/mL), but TPZ-I (10 μg/mL and 5 μg/mL) was more effective in enhancing GSH-px production.

#### 2.6.3. Effects of Acidic Polysaccharides on the Activities of T-AOC and ROS

**Figure 5:**
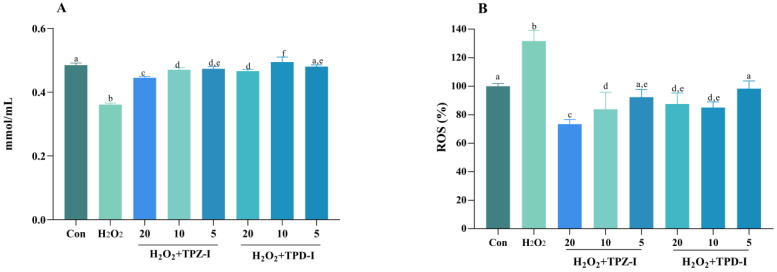
Effects of TPZ-I and TPD-I on the activities of total antioxidant capacity-T-AOC (**A**), and reactive oxygen species-ROS (**B**) in IPEC-J2 cells. The concentrations of the polysaccharide fractions were 20, 10, and 5 μg/mL, respectively. Values are presented as means ± SD (*n* = 6). Bars with different letters are statistically significant (*p* < 0.05).

The effects of TPZ-I and TPD-I on the activities of total antioxidant capacity (T-AOC) and reactive oxygen species (ROS) in IPEC-J2 cells are shown in Figure 5. From Figure 5, the two acidic polysaccharide fractions increased the total antioxidant capacity (T-AOC) of IPEC-J2 cells. The acidic polysaccharides from *T. pseudoexcavatum* (TPD-I) had a greater effect on T-AOC, especially at a dose of 10 μg/mL. The 20 μM H_2_O_2_ treatment increased the ROS level compared to the control group, yet the two polysaccharides significantly decreased the ROS level and reduced the damage of H_2_O_2_ to cells (*p* < 0.05).

In this study, the differences in antioxidant activity between two acidic polysaccharides from different truffle species were investigated, and different biological activities were identified. Biological activity was related to the structure of the polysaccharides, including monosaccharide composition, molecular weight, and glycoside linkage. Several studies showed that higher glucose content in the polysaccharide could lead to higher antioxidant activity [27,28], which was consistent with fraction TPD-I and TPZ-I. TPD-I contains greater glucose contents than TPZ-I, leading to higher antioxidant activity. Moreover, the monosaccharide composition of Gal also affected the antioxidant activity. There is evidence to prove that higher Gal content leads to relatively higher antioxidant activity [29,30]. However, the fraction TPZ-II showed no obvious activity, and it contains a higher content of glucose and galactose than TPZ-I, but lower content of glucose and galactose than TPD-I. This indicated that the content of glucose and galactose alone is not enough to judge the antioxidant activity of polysaccharides. Additionally, there is a link between the lower molecular weight of a polysaccharide and greater antioxidant activity [14,31,32,33]. In the present study, apart from the monosaccharide composition of TPZ-I and TPD-I, the biggest structural difference is the molecular weight. The molecular weight of TPD-I is much lower than that of TPZ-I, which leads to different antioxidant activities of the polysaccharides. The exception case here is also the fraction TPZ-II, which has a similar molecular weight to TPD-I but showed no obvious activity. Therefore, differences in monosaccharide content (glucose and galactose) and molecular weight may lead to differences in antioxidant activity, but not the determinant. Studies related to the structure-activity relationship need to be carried out further.

## 3. Materials and Methods 

*T. panzhihuanense* and *T. Pseudoexcavatum* were collected from Yongren County, Yunnan Province, China. The voucher specimen was deposited at the Sichuan Academy of Agricultural Sciences (Voucher no. 20190407).

### 3.1. Extraction of Polysaccharides

*Tubers* were weighed (200 g) and dried. After removing lipophilic and low molecular compounds with 95% ethanol, the truffles were boiled in water to obtain the crude water extracts. The concentrated crude extracts were first dialyzed using a cut-off of 3500 Da, and precipitated by using 4 folds ethanol at 4 °C overnight. The precipitates were collected and lyophilized. The crude polysaccharide fraction from *T. panzhihuanense* was named TPZ, while the crude polysaccharide extract from *T. pseudoexcavatum* was named as TPD.

The determination of carbohydrate content in polysaccharides was performed as previously described, using phenol-sulfuric acid [34]. Meanwhile, the polyphenol content was measured by the Folin-Ciocalteu method, and gallic acid was used as the standard [35]. The content of protein was quantified based on the Bradford method, and the standard was bovine serum albumin [36].

### 3.2. Purification of Polysaccharides

#### 3.2.1. Ion-Exchange Chromatography on Diethylaminoethyl-Cellulose

Two crude polysaccharides (300 mg) were dissolved in deionized water (20 mL), respectively. The ion-exchange chromatograph was equipped with a 50 × 600 mm DEAE Sepharose Fast Flow column for polysaccharide separation. The two polysaccharide solutions were each filtered through a 0.45 μm membrane and injected onto the column. The elution programs were as follows: 2 mL/min deionized water (neutral fractions); 2 mL/min linear NaCl gradient in water (0–1.5 M) (acidic fractions), which were monitored using the phenol-sulfuric acid method [37]. The acidic carbohydrate fractions were dialyzed against distilled water at a cut-off of 3500 Da. The dialyzed fractions were concentrated and lyophilized.

#### 3.2.2. Acidic Polysaccharide Purification

Gel filtration chromatography was used to purify the acidic polysaccharide fractions. Twenty milligrams of acidic polysaccharide from Section 3.2.1. was dissolved in 10 mM sodium chloride solution and filtered through a 0.45 μm membrane. The solution was loaded onto the Sepharose 6FF column and eluted with 1.0 mL/min NaCl (10 mM). The polysaccharide fractions were pooled according to the elution profile, and purified fractions were finally obtained after the dialysis and lyophilization.

### 3.3. Determination of Molecular Weight

Size exclusion chromatography (SEC) with a Hiload^TM^ 16/60 Superdex^TM^200 prep grade column (General Healthcare, Uppsala, Sweden) was used to measure the polysaccharides molecular weight. The elution solvent was distilled water, at a flow rate of 0.4 mL/min. The column was calibrated with standard dextran to obtain a standard curve [38]. The standard curve was used to calculate the relative *Mw* of the polysaccharide fractions.

### 3.4. Chemical Compositions and Glycosidic Linkage Determination

The monosaccharide compositions of the polysaccharides were determined by gas chromatography. The trimethylsilylated derivative of methyl glycoside was reacted with hydrochloride acid (3 mol/L) in anhydrous methanol for 24 h at 80 °C. After methanolysis, the fractions were analyzed by capillary GC (Thermo Scientific, Milan, Italy), with mannitol as the internal standard. The GC conditions followed previously published methods [39].

Meanwhile, the elucidation of glycosidic linkages was determined by methylation. Before methylation, free uronic acid was reduced to the neutral sugar NaBD_4_. After reduction of the polymer, methylation, hydrolysis, reduction, and acetylation were carried out [40]. A GCMS-QP2010 was used to analyze the partly acetylated and partly methylated alditols (Shimadzu, Kyoto, Japan), and the compound corresponding to each peak was characterized by the interpretation of the retention times and the characteristic mass spectra. The relative amounts of each linkage type were estimated by the total amount of each monosaccharide type [41].

### 3.5. Antioxidant Activity Assay

In this study, all the five polysaccharide fractions were tested for cell viability first, and the fractions that showed obvious activity were chosen for further study. The acidic fraction TPZ-II was not chosen for further antioxidant activity examination as it showed no obvious activity.

#### 3.5.1. Cell Culture

IPEC-J2 cells (intestinal porcine epithelial cell lines) were maintained in DMEM/F-12 medium in a humidified atmosphere of 5% CO_2_ at 37 °C. The medium contained 100 U/mL streptomycin, 100 U/mL penicillin, and 10% fetal bovine serum (FBS).

#### 3.5.2. Establishment of an Oxidative Stress Damage Model

IPEC-J2 cells were seeded into 96-well plates at a density of 1.0 × 10^4^ cells/well. After the cells adhered to the 96-well plate, the cells were washed with phosphate buffered saline (PBS, pH = 7.4) three times. Next, 200 μmol/mL of H_2_O_2_ (Sigma-Aldrich, St. Louis, MO, USA) was added to each well (*n* = 12), and the cells were cultured in a 37 °C incubator. After 24 h of incubation, 10 μL cell counting kit-8 (CCK8) (Wuhan Boster Biological Technology., Ltd., Wuhan, China) was added to each well, and after 1 h incubation, the absorbance was measured at 450 nm using a microwell reader (Bio-Rad, Software Version 8.0) [42].

#### 3.5.3. Measurement of IPEC-J2 Cells Viability

IPEC-J2 cells seeded onto 96-well plates were cultured in a 37 °C incubator for 24 h. Then, 200 μmol/mL H_2_O_2_ was added to each well, and the cells were cultured for 24 h. Three concentrations of each polysaccharide fraction (5 μg/mL, 10 μg/mL, 20 μg/mL) were added to the 96-well plate. The cell viability was determined by the CCK8 method after incubation for 24 h at 37 °C [42].

#### 3.5.4. Determination of Antioxidant Enzymes Activity

IPEC-J2 cells were seeded onto a six-well plate, and an oxidative stress damage model was established using 200 μmol/mL of H_2_O_2_. Different concentrations of the tested polysaccharide fractions (5 μg/mL, 10 μg/mL, 20 μg/mL) were added to the six-well plate. After 24 h culture at 37 °C, plates were washed with PBS three times. Cells were collected from the cell culture dish and disrupted using a cell ultrasonic cell breaker (Huxi Industry Co., Ltd., Shanghai, China). Cells were centrifuged at 12,000 rpm for 3 min after cell disruption to obtain the supernatant for the determination of antioxidant enzyme activity. The antioxidant enzyme activities were determined by a Biochemical Detection Kit (Nanjing Jiancheng Bioengineering Institute, Nanjing, China) [42].

### 3.6. Statistical Analysis

The mean and standard deviation of the data were calculated, and one-way analysis of variance (ANOVA) by Duncan’s test was performed for statistical comparisons. The SPSS (v20.0) software was used for statistical analysis. Values at *p* < 0.05 were considered statistically significant.

## 4. Conclusions

In summary, five polysaccharide fractions were purified from *T. panzhihuanense* and *T. pseudoexcavatum* (TPZ-NP, TPZ-I, TPZ-II, TPD-NP, and TPD-I), and the molecular weights were 5.13, 30.84, 17.43, 8.37, and 18.91 kDa, respectively. The monosaccharide compositions and glycosidic units were determined, and the results showed similar monosaccharide compositions among the fractions but different molar ratios. The antioxidant activities of two acidic polysaccharides, TPZ-I and TPD-I, were also investigated. The results showed that both improved the viability of IPEC-J2 cells and increased the content of SOD, T-AOC, GSH-px, and CAT in cells. Moreover, TPZ-I and TPD-I inhibited the production of MDA and ROS stimulated by oxidative stress, thus showing certain antioxidant effects. TPD-I exhibited better antioxidant activity than TPZ-I, which may be due to the difference in structure. However, further studies need to be focused on the detailed structure-activity relationship. 

## Figures and Tables

**Table 1:** The content determination of crude polysaccharide from *Tubers*.

	TPD	TPZ
Protein	3.79%	4.15%
Phenol	10.11%	15.48%
Carbohydrates	57.90%	67.40%

**Table 2:** The molecular weight of polysaccharides from two truffle species.

Fraction	Molecular Weight (kDa)
TPZ-NP	5.13
TPZ-I	30.84
TPZ-II	17.43
TPD-NP	8.37
TPD-I	18.91

**Table 3:** The monosaccharide compositions (mol%) of polysaccharide fractions isolated from *T. panzhihuanense* and *T. pseudoexcavatum.*

	TPZ-NP	TPZ-I	TPZ-II	TPD-NP	TPD-I
Rha ^a^	23.9	23.0	22.2	19.8	19.7
Man ^b^	62.3	67.8	58.8	63.4	56.0
Gal ^c^	4.7	3.6	5.6	2.4	4.7
Glc ^d^	9.2	4.8	9.4	14.4	17.7
ManA ^e^	n.d. ^f^	0.9	4.0	n.d. ^f^	1.9

Note: ^a^ Rhamnose, ^b^ Mannose, ^c^ Galactose, ^d^ Glucose, ^e^ Mannuronic acid, ^f^ not detected. TPZ-NP, TPZ-I, and TPZ-II are purified from *T. panzhihuanense*; TPD-NP and TPD-I are purified from *T. pseudoexcavatum*.

**Table 4:** The glycosidic linkages of polysaccharide fractions from truffles.

		TPZ-I	TPD-I
Rha	T	12.6	9.7
	1→2	5.4	5.6
	1→3	5.0	4.4
Man	T	5.2	8.0
	1→2	23.3	15.6
	1→3	18.7	12.4
	1→6	2.3	1.8
	1→2, 3	3.0	3.6
	1→3, 6	1.9	n.d.
	1→2, 6	13.4	14.6
ManA	1→4	0.9	1.9
Gal	T	1.3	1.4
	1→3	2.3	3.3
Glc	1→4	4.8	17.7
